# Vertical Phase Regulation with 1,3,5‐Tribromobenzene Leads to 18.5% Efficiency Binary Organic Solar Cells

**DOI:** 10.1002/advs.202303150

**Published:** 2023-07-09

**Authors:** Chaofeng Zhu, Sein Chung, Jingjing Zhao, Yuqing Sun, Bin Zhao, Zhenmin Zhao, Seunghyun Kim, Kilwon Cho, Zhipeng Kan

**Affiliations:** ^1^ Center on Nanoenergy Research Guangxi Colleges and Universities Key Laboratory of Blue Energy and Systems Integration Carbon Peak and Neutrality Science and Technology Development Institute School of Physical Science & Technology Guangxi University Nanning 530004 China; ^2^ Department of Chemical Engineering Pohang University of Science and Technology 77 Cheongam‐ro, Nam‐gu Pohang‐si 37673 South Korea; ^3^ State Key Laboratory of Featured Metal Materials and Life‐cycle Safety for Composite Structures Nanning 530004 China

**Keywords:** bimolecular recombination, sequential deposition, vertical phase distribution, volatile solid additives

## Abstract

The sequential deposition method assists the vertical phase distribution in the photoactive layer of organic solar cells, enhancing power conversion efficiencies. With this film coating approach, the morphology of both layers can be fine‐tuned with high boiling solvent additives, as frequently applied in one‐step casting films. However, introducing liquid additives can compromise the morphological stability of the devices due to the solvent residuals. Herein, 1,3,5‐tribromobenzene (TBB) with high volatility and low cost, is used as a solid additive in the acceptor solution and combined thermal annealing to regulate the vertical phase in organic solar cells composed of D18‐Cl/L8‐BO. Compared to the control cells, the devices treated with TBB and those that underwent additional thermal processing exhibit increased exciton generation rate, charge carrier mobility, charge carrier lifetime, and reduced bimolecular charge recombination. As a result, the TBB‐treated organic solar cells achieve a champion power conversion efficiency of 18.5% (18.1% averaged), one of the highest efficiencies in binary organic solar cells with open circuit voltage exceeding 900 mV. This study ascribes the advanced device performance to the gradient‐distributed donor‐acceptor concentrations in the vertical direction. The findings provide guidelines for optimizing the morphology of the sequentially deposited top layer to achieve high‐performance organic solar cells.

## Graphical abstract

Solid additive 1,3,5‐tribromobenzene (TBB) with centrosymmetric structure shows excellent potential in optimizing molecular aggregation and stacking, tuning the donor‐acceptor distribution in the vertical direction, and improving the device stability. In tandem with the synergistic effect of the sequential deposition processing method and thermal treatment, a champion power conversion efficiency of 18.5% is achieved with D18‐Cl/L8BO as the photoactive layer.

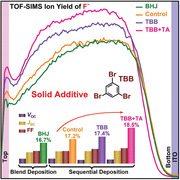

## 1 Introduction

Solution‐processed organic solar cells (OSCs) attract broad research interest for their low cost, lightweight, environmental friendliness, and capability to fabricate large‐area devices on flexible substrates via roll‐to‐roll technology.^[^ ^1^, ^2^, ^3^, ^4^, ^5^, ^6^, ^7^, ^8^, ^9^ ^]^ The state‐of‐the‐art OSCs composed of PM6:Y6 and their derivatives constantly achieve power conversion efficiencies (PCEs) of over 15% for single‐junction devices.^[^ ^10^, ^11^, ^12^, ^13^, ^14^, ^15^, ^16^ ^]^ Recently, combined with acceptor synthesis and a quaternary strategy, the OSCs with PCE of 19.76% were reported, originating from the better compromise between charge generation and recombination regulated by the BTP‐S17 and BTP‐S16 mixture.^[^ ^17^ ^]^ Therefore, managing the nanoscale bi‐continuous donor‐acceptor interpenetrating network in the photoactive layer to optimize the charge dynamics for high‐performance OSCs remains challenging.

A two‐step sequential deposition (SD) of the donor and acceptor in the photoactive layer and processing additives are proven effective in efficiently regulating the vertical distribution of the donor and acceptor layer.^[^ ^18^, ^19^, ^20^ ^]^ In contrast to the one‐step deposition method, the sequential thin film deposition follows a layer‐by‐layer (LBL) casting process from two separated solutions with similar/same solvent, forming a pseudo‐bilayer configuration in the photoactive layer.^[^ ^21^, ^22^, ^23^ ^]^ The pseudo‐bilayer structure differs from the true “bilayer” processed with orthogonal solvents to avoid erosion of the layer beneath.^[^ ^24^ ^]^ For instance, the OSCs composed of D18 and L8‐BO were fabricated with the LBL casting method, and by optimizing the spin‐coating conditions, the vertical phase separation was optimized, leading to a PCE of 19.05%.^[^ ^25^ ^]^ The impact of the solvent additive, 1‐chloronaphthalene (CN), on the charge dynamics was analyzed by fabricating OSCs composed of PM6/Y6 with the LBL thin film coating process. The ultrafast spectroscopy results showed that adding 0.5 vol.% (volume percentage) CN in the Y6 solution could facilitate exciton dissociation and charge separation. In contrast, excessive (> 1 vol.%) use of CN causes fast geminate and non‐geminate charge recombination, leading to poor device performance.^[^ ^26^ ^]^ Adding 0.25 vol.% 1,8‐diiodooctane (DIO) and different weight ratios of BTP‐S2 in BO4Cl solution, the photoactive layer composed of PM6/(BO4Cl:BTPS2) could form a morphology with donor‐enrichment at the anode and acceptor‐enrichment at the cathode prepared with LBL method. The reduced charge recombination and promoted charge transport/collection properties led to an improved fill factor (FF) of 78.04%, an enhanced short circuit current density (*J*_SC_) of 27.14 mA cm^−2^, and a PCE of 18.16%.^[^ ^27^ ^]^ It is worth noting that the high boiling point (bp.) liquid additives (e.g., DIO with a bp. of 332.5 °C and CN with a bp. of 260.3 °C) are usually difficult to remove completely,^[^ ^1^, ^28^, ^29^ ^]^ resulting in unstable photoactive layer morphologies and poor device reproducibility.^[^ ^30^, ^31^ ^]^ To overcome this drawback, volatile solid additives that could evaporate entirely from the photoactive layer become a practical approach to optimizing the photoactive layer. It was reported that 1,3‐dibromo‐5‐chlorobenzene^[^ ^32^, ^33^ ^]^ and 1,4‐diiodobenzene^[^ ^34^, ^35^, ^36^ ^]^ could help acceptor molecules to form tighter molecular packing, enhance the intermolecular π–π stacking, and more ordered microstructures for the non‐fullerene acceptor, resulting in high‐performed OSCs. However, low prices and efficient volatile solid additives still lag behind the development of non‐fullerene OSCs.

In this contribution, we systematically investigated the impact of a novel volatile solid additive, 1,3,5‐tribromobenzene (TBB), on the vertical phase segregation of LBL‐processed D18‐Cl/L8‐BO. TBB was added to the chloroform (CF) solution of L8‐BO, and it can be completely evaporated from the photoactive layer by thermal annealing (TA) at 75 °C for five minutes. The OSCs composed of D18‐Cl/L8‐BO with TBB and thermal treatment exhibited a high open circuit voltage (*V*_OC_) of 910 mV, a *J*_SC_ of 26.3 mA cm^−2^, an FF of 77.2%, and a PCE of 18.5%, which is higher than the control devices (17.2%). To our knowledge, the *V*_OC_ achieved here is one of the highest in OSCs with >18% efficiency. The improved device performance was ascribed to the enhanced exciton dissociation rate, charge mobility, and reduced charge recombination, originating from the distribution of the acceptor‐donor in the vertical direction and the increased thin film crystallinity. Our results provide alternative design guidelines on volatile solid additives for high‐performing non‐fullerene OSCs.

## 2 Results and Discussion

**Figure 1:**
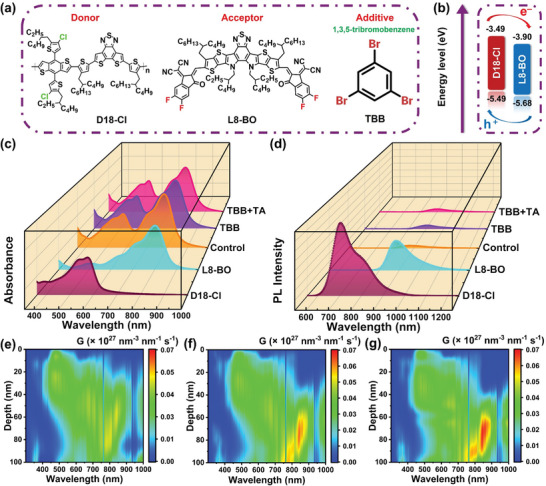
a) Chemical structures of D18‐Cl, L8‐BO, and 1,3,5‐tribromobenzene (TBB). b) Energy level diagrams of D18‐Cl and L8‐BO. c) Absorbance and d) photoluminescence spectra of the neat and LBL‐processed films. Exciton generation rates at each wavelength as a function of the film thickness of the LBL‐processed e) D18‐Cl/L8‐BO (Control), f) D18‐Cl/L8‐BO (TBB), and g) D18‐Cl/L8‐BO (TBB+TA).

The chemical structures of D18‐Cl, L8‐BO, and TBB are shown in **Figure** **1**a. The highest occupied molecular orbitals (HOMO) and lowest unoccupied molecular orbitals (LUMO) of D18‐Cl and L8‐BO were taken from reported values,^[^ ^14^, ^16^, ^35^, ^37^ ^]^ as shown in Figure 1b. The energy difference between the HOMO of D18‐Cl and LUMO of the L8‐BO is larger than that between PM6 and Y6, the state‐of‐the‐art photoactive layer combination, indicating that the potential *V*_OC_ could be >840 mV.^[^ ^4^, ^32^, ^36^, ^38^ ^]^ As presented in Figure 1c, two absorption peaks at 576 and 803 nm correspond to the maximum absorption of neat D18‐Cl and L8‐BO films, respectively. Compared with the control film, the TBB‐processed (TBB) and TBB‐processed in combination with TA (TBB+TA) samples exhibit red‐shifted spectra in the absorption range of L8‐BO, indicating the molecular packing of L8‐BO was influenced by introducing TBB. The enlarged differences in spectra are provided in Figure S2 (Supporting Information). Notably, TBB has been entirely evaporated from the thin films, as evidenced by the Fourier‐transform infrared (FTIR) spectroscopy shown in Figure S3c (Supporting Information), in which the distinct functional group (C‐Br) peak of TBB located at 655 cm^−1^ disappears in the TBB and the TBB+TA films. The photoluminescence (PL) spectra of the neat D18‐Cl and L8‐BO films are plotted in Figure 1d. When L8‐BO was photoexcited, comparable PL quenching efficiency of 86.2%, 85.9%, and 87.1% for the control, TBB, and TBB+TA samples were obtained, respectively. On the other hand, when the neat D18‐Cl was photoexcited, the PL quenching efficiency was close to unity in all the thin films, implying efficient exciton dissociations. As illustrated in Figure 1e–g, transfer matrix simulation was employed to estimate the exciton generation rate with a conventional device architecture of ITO/PEDOT:PSS/active layer/PDIN/Ag. The observations indicate that the exciton generation rate remains unchanged across all devices within the D18‐Cl absorption range. However, in the L8‐BO absorption range, the addition of TBB and TBB+TA leads to a significant increase in the exciton generation rate, implying an enhanced contribution to photocurrent conversions of L8‐BO.

**Figure 2:**
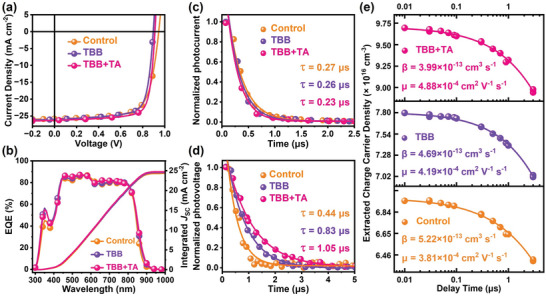
a) *J–V* characteristics, b) external quantum efficiency (EQE) spectra, c) transient photocurrent, d) transient photovoltage, and e) charge carrier density versus delay time of the Control, TBB, and TBB+TA devices composed of D18‐Cl/L8‐BO. The solid lines in (c), (d), and (e) fit to the experimental data.

We fabricated conventional OSCs to check the performance variations of the control, TBB, and TBB+TA devices, and the OSCs were measured under AM 1.5G simulated solar light with 100 mW cm^−2^ intensity. The *J–V* characteristics are plotted in **Figure** **2**a, and the photovoltaic parameters of all the OSCs are summarized in **Table** **1**. The control OSCs exhibit a PCE of 17.2% with a *J*_SC_ of 25.8 mA cm^−2^, an FF of 70.6%, and a *V*_OC_ of 945 mV. When TBB was added to the CF solution of L8‐BO, the resulting *J*_SC_ and FF were enhanced, but the *V*_OC_ decreased, resulting in a PCE of 17.4%. Remarkably, adding TBB in the device and combining it with TA (at 75 °C for five minutes) further promoted the *J*_SC_ and FF, and a champion PCE of 18.5% was achieved, associated with a *V*_OC_ of 910 mV, a *J*_SC_ of 26.3 mA cm^−2^, and an FF of 77.2%. To our knowledge, the *V*_OC_ (910 mV) is among the highest for OSCs, achieving an efficiency of over 18%. The external quantum efficiency (EQE) spectra of the control, TBB, and TBB+TA devices are presented in Figure 2b. The EQE of TBB devices shows improvements in ca. 350–450, 450–650, and 650–950 nm. Especially the increment in the L8‐BO absorption range proved that a fast exciton generation rate could lead to higher photocurrent conversions. The *J*_SC_ integrated from EQE is within a 5−7% deviation compared with the *J*_SC_ measured with a solar simulator. Additionally, the *J–V* curves under the dark are plotted in Figure S4 (Supporting Information), and we fitted the positive bias part with the diode equation (Table S2, Supporting Information). The shunt resistance *R*_sh_ values, which determine current leakage, were 2.5 × 10^4^, 8.4 × 10^4^, and 20.5 × 10^4^ Ω cm^2^ for the control, TBB, and TBB+TA devices, respectively. Additionally, ideality factor *n* values of 1.57, 1.70, and 1.55 were obtained for the control, TBB, and TBB+TA devices, respectively. These values suggest the presence of traps in the devices, with the TBB devices exhibiting a more significant degree of trap‐assisted charge recombination.

**Table 1:** Parameters of the Control, TBB, and TBB+TA OSCs under AM 1.5 G simulated irradiance (100 mW cm^−2^). Average values with standard deviation (in parenthesis) were obtained from 20 independent devices. The calculated *J*_SC_ values listed were integrated from the EQE spectra

Conditions	*V*_OC_ [mV]	*J*_SC_ [mA cm^−2^]	*J*_cal, EQE_ [mA cm^−2^]	FF [%]	PCE [%]
Control	945 (939 ± 3)	25.8 (25.2 ± 0.4)	24.3	70.6 (70.3 ± 0.4)	17.2 (16.7 ± 0.3)
TBB	895 (910 ± 9)	26.0 (25.6 ± 0.3)	24.6	74.8 (74.2 ± 0.8)	17.4 (17.2 ± 0.2)
TBB+TA	910 (900 ± 8)	26.3 (26.4 ± 0.4)	24.8	77.2 (76.2 ± 0.8)	18.5 (18.1 ± 0.3)

The impact of TBB solid additive and TA on charge carrier dynamics was investigated. Figure 2c displays the transient photocurrent decay, and the charge extraction time of 0.27, 0.26, and 0.23 µs are fitted for the control, TBB, and TBB+TA devices, respectively. The resulting extraction time indicates that adding TBB and further TA treatment could effectively facilitate the extraction of charge carriers. The charge carrier lifetimes at open‐circuit conditions were extracted from the transient photovoltage decay dynamics by fitting them to a mono‐exponential model presented in Figure 2d. The TBB+TA device exhibits a carrier lifetime value of 1.05 µs, longer than the control and TBB counterparts (0.44 and 0.83 µs), implying reduced charge recombination in TBB+TA devices due to the positive morphological changes. To get the charge carrier mobility and the bimolecular charge recombination rate, we performed the time‐delayed photon‐induced charge‐carrier extraction in linearly increasing voltage (photo‐CELIV). The faster charge carrier mobility (*µ*) of the devices can be calculated with the equation of $\mu =\frac{2d^{2}}{3At_{\max}^{2}\left(1\;+\;0.36\Delta j/j_{0}\right)}$, where *d* is the active layer thickness, *A* is the ramp rate, *t*_max_ is the time when the extracted current reaches its maximum value, *j*_0_ represents the displacement current, and *Δ*j is the transient current peak height.^[^ ^39^, ^40^, ^41^ ^]^ The mobility values of 3.81 × 10^−4^ cm^2^ V^−1^ s^−1^ for the control devices, 4.19 × 10^−4^ cm^2^ V^−1^ s^−1^ for the TBB devices, and 4.88 × 10^−4^ cm^2^ V^−1^ s^−1^ for the TBB+TA devices are obtained. The apparent higher mobility suggests the positive impact on the active layer morphology caused by the addition of TBB and TA. Besides the faster charge mobility, the time‐resolved charge carrier density integrated from the current transients follows the bimolecular charge recombination behavior in the OSCs. Thus, we fitted the extracted charge carrier density in Figure 2e to get the bimolecular charge recombination rate (the notes on the bimolecular recombination fitting are presented in Section 7, and the current transient of photo‐CELIV measurements are plotted in Figure S5, Supporting Information). The bimolecular charge recombination rates *β* (Table S3, Supporting Information) of the control, TBB, and TBB+TA devices are 5.22 × 10^−13^ cm^3^ s^−1^, 4.69 × 10^−13^ cm^3^ s^−1^, and 3.99 × 10^−13^ cm^3^ s^−1^, respectively. The smaller *β* means slower bimolecular charge recombination, which aligns with the transient photovoltage measurements. So far, the device performance and charge dynamics have been analyzed quantitatively. Now we turn to discuss their morphological origins.

**Figure 3:**
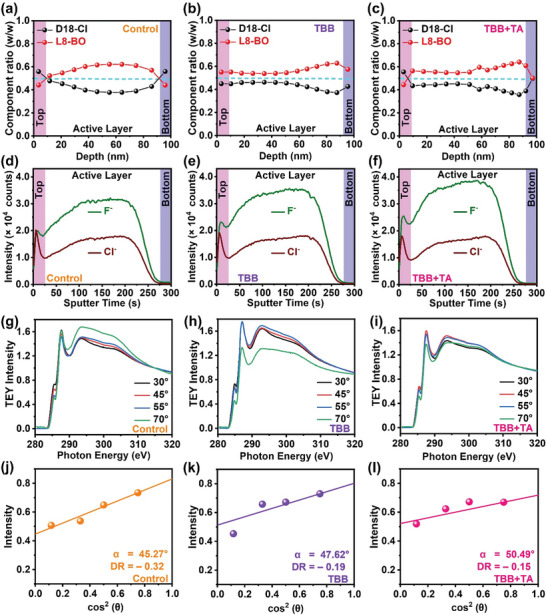
The composition profiles extracted from the film‐depth‐dependent light absorption spectroscopy (FLAS) spectra of the a) Control, b) TBB, and c) TBB+TA films. Time‐of‐fight secondary ion mass spectrometry (TOF‐SIMS) ion yield of F^−^ and Cl^−^ as a function of sputtering time for the d) Control, e) TBB, and f) TBB+TA–films composed of D18‐Cl and L8‐BO. Angular dependence of near‐edge X‐ray absorption fine‐structure (NEXAFS) spectra with X‐ray beam at 30°, 45°, 55°, and 70° in total electron yield (TEY) detection mode of the g) Control, h) TBB, and i) TBB+TA films, respectively. A plot of the intensity of the π^*^ manifold according to angles with surface molecular orientation analysis of the j) Control, k) TBB, and l) TBB+TA films.

We used film‐depth‐dependent light absorption spectroscopy (FLAS) and time‐of‐flight secondary ion mass spectrometry (TOF‐SIMS) to investigate the vertical phase segregation in thin films. Figure S6a–c (Supporting Information) show that both D18‐Cl and L8‐BO absorption peaks are present throughout the etched films in the control, TBB, and TBB+TA samples, indicating the formation of a bulk heterojunction (BHJ) active layer structure through the penetration of L8‐BO into the D18‐Cl layer during spin‐coating. The absorption peaks of L8‐BO exhibit a red shift upon TBB and TA treatments, suggesting the presence of molecular packing and energy landscape heterogeneity along the film depth. Besides the film structural information, the composition of the D18‐Cl and L8‐BO at different film‐depth could be extracted, as depicted in Figure 3a–c. The penetration of the L8‐BO solution leads to an initial increase in the L8‐BO weight ratio at a depth of ≈60 nm, followed by a reduction probably caused by the thin film drying process (**Figure** **3**a). After the addition of TBB (Figure 3b), the donor‐acceptor ratio remains stable at 55.5 wt.% (weight percentage) L8‐BO and 44.5 wt.% D18‐Cl up to a depth of ≈50 nm, after that the L8‐BO ratio gradually increases toward the bottom of the thin film, resulting in acceptor enrichment near the anode. Upon thermal treatment, the composition of donor and acceptor is similar to that of the TBB samples; however, the L8‐BO load at the bottom is equivalent to that of D18‐Cl (Figure 3c), which could potentially suppress bimolecular charge recombination by reducing the L8‐BO accumulation.

The fluorine (L8‐BO) and chlorine (D18‐Cl) ions, F^−^ and Cl^−^, were traced with the TOF‐SIMS measurements. Both ions' intensities in the vertical direction as a function of the sputtering time are illustrated in Figure 3d–f for the control, TBB, and TBB+TA films, respectively. The higher F^−^ intensity compared to Cl^−^ suggests that the acceptor concentration is higher than that of the donor, which agrees with the FLAS results. Besides, a stronger F^−^ signal on top of the active layer indicates L8‐BO rich phase present on the top. As a comparison, the F^−^ and Cl^−^ intensity in a BHJ active layer is provided in Figure S7e (Supporting Information). With the increasing sputtering time (etching depth), the intensities of the F^−^ signal for the TBB+TA and the TBB films are still stronger than the control and the BHJ‐film presented in Figure S7f (Supporting Information), implying L8‐BO enriches more on the shallow layer of the LBL samples. In contrast, the intensity of the F^−^ signal in the TBB+TA film at the bottom of the active layer is lower than those of other films, implying the D18‐Cl enriches at the bottom layer. Though the donor‐acceptor weight ratio is not easy to estimate from the ions' intensity, these results provide fundamental proof that with the assistance of TBB and thermal treatment, the vertical phase separation could be optimized, and a graded donor‐acceptor morphology could form to reduce the charge recombination and improve charge transport.

Likewise, the vertical phase compositions at the thin film surface and the out‐of‐plane molecular orientation structure of the highly ordered surface can be accessed with near‐edge X‐ray absorption fine‐structure (NEXAFS) spectroscopic characterizations. The NEXAFS spectroscopy is a technique that examines the X‐ray absorption spectrum of a material near one of its absorption edges. We performed NEXAFS spectroscopy in the energetic range of 280–320 eV to analyze the K‐edge band of carbon by tilting the angles. Tuning the angles in NEXAFS spectroscopy can differentiate the degree of pi‐orbital overlapping, transition dipole moments (TDM), which is oriented perpendicular to the conjugated ring plane so that could provide information about the average tilt angle of the conjugated backbones.^[^ ^42^ ^]^ Figure 3g–i shows the angle‐resolved NEXAFS (30°, 45°, 55°, and 70°) spectra of the control, TBB, and TBB+TA blend film with total electron yield (TEY) modes that have a surface sensitivity of 3 nm.^[^ ^43^ ^]^ The uppermost layers of the control, TBB, and TBB+TA thin‐film were finely angle‐resolved, and the equally first observed peak of the 285.5 eV indicates the 1s → π^*^ (C═C) transitions. The intensity of the π^*^ manifold as a function of the X‐ray angles of incidence is illustrated in Figure 3j–l, and the plotted data is fitted with the expression presented below:

(1) $$I=\frac{1}{3}\left[1+\frac{1}{2}\left(3\cos^{2}\theta -1\right)\left(3\cos^{2}\gamma -1\right)\right]$$

where *I* is the total electron yield intensity, *θ* is the angle of X‐ray incidence, and *γ* is the average tilted angle of the TDM. For TEY detection, an average tilted angle α of the conjugated backbone is 45.27° (*γ* = 44.73°), 47.62° (*γ* = 42.38°), and 50.49° (*γ* = 39.51°) is found on the control, TBB, TBB+TA thin‐film, respectively. To deconvolution the interaction of D18‐Cl and L8‐BO, we performed the NEXAFS spectra on the neat films, respectively. As shown in Figure S8a–d (Supporting Information), L8‐BO presents a perfect face‐on orientation upon film surfaces with a dichroic ratio, DR = (*A*_⊥_ − *A*_∥_)/(*A*_⊥_ + *A*_∥_), of −0.98. In contrast, the D18‐Cl donor has a slightly tilted backbone with an angle of 41.30°(DR = −0.44) from the substrate. After the addition of the TBB additive into the control film, the D18‐Cl slightly promotes raising the average tilted angle through a mutual diffusion process into the L8‐BO abundant upmost part of the thin film. Moreover, the thermal treatment of TBB‐added thin film clearly accelerates the diffusion process mentioned above with optimized morphology. The analysis on the NEXAFS spectra could provide evidence that the donor and acceptor are much more interpenetrating with each other during the SD processing method.

**Figure 4:**
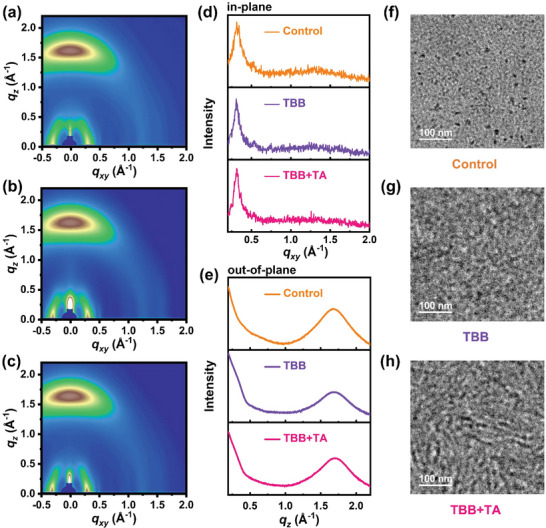
2D GIWAXS patterns of the blend films: the a) Control, b) TBB, and c) TBB+TA. GIWAXS intensity profiles of the blend films along the corresponding d) in‐plane and e) out‐of‐plane line cuts. TEM images of the blend films: the f) Control, g) TBB, and h) TBB+TA.

Grazing incidence wide‐angle X‐ray scattering (GIWAXS) measurement was employed to analyze the variation of crystallinity and molecular packing behaviors of the thin films. Figure 4a–c shows the 2D GIWAXS patterns of the control, TBB, and TBB+TA films, while Figure S9a,b (Supporting Information) present the patterns of neat D18‐Cl and L8‐BO, respectively. The information on the in‐plane (IP) and out‐of‐plane (OOP) parameters, such as peak position, d‐spacing, full width at half maximum (FWHM), and crystal coherence length (CCL) extracted from the 2D GIWAXS of the five films are described in Table S4 in Section 13 (Supporting Information). The (010) diffraction peaks appear in the OOP direction and correspond to the π–π stacking, which indicates their preferred face‐on packing nature. Both donor and acceptor show good crystalline order in neat films (Figure S9a,b, Supporting Information). In the blend films, the (010) π–π stacking peaks are observed at *q*_z_ = 1.677, 1.684, and 1.697 Å^−1^ for the control, TBB, and TBB+TA films, with corresponding d‐spacing values of 3.75, 3.73, and 3.70 Å, respectively. In the blend films, conjugated D18‐Cl and L8‐BO are preferentially face‐on oriented according to π–π stacking peak locations, and the observed trend of increased q values indicates tighter π–π stacking distance, facilitating charge carrier transport properties. The CCL is derived from the Scherrer equation: CCL = 2π × 0.9/FWHM to analyze the molecular stacking in blend films. The resulting CCLs for the control, TBB, and TBB+TA films are 20.39, 19.61, and 20.96 Å, respectively. Compared to the control films, the larger CCLs in the solid additive TBB‐treated films demonstrate that the solid additive can effectively enhance the crystallinity and phase separation in the thin films. The TBB+TA films exhibit the highest crystallinity and form a bi‐continuous donor‐acceptor network, consistent with the observed experimental increase in mobility and decreased charge carrier recombination.

In addition, we conducted atomic force microscopy (AFM) and transmission electron microscopy (TEM) measurements to visualize the bulk morphology of the photoactive layer. The root‐mean‐square (RMS) roughness values are 0.66, 0.98, and 1.15 nm for the control, TBB, and TBB+TA blend films (Figure S10d–f, Supporting Information), respectively. The changes in the surface roughness provide the crystalline characteristics of the layers beneath, with larger values indicating greatly enhanced thin film crystallinity.^[^ ^34^, ^35^, ^36^, ^44^ ^]^ These observations are in line with the TEM results presented in Figure 4f–h. The control films exhibit well‐mixed domains^[^ ^45^ ^]^ resulting from the penetration of the upper layer solution (**Figure** **4**f). However, the addition of TBB and further TA gradually increase molecular aggregation features (Figure 4g,h). Notably, the TBB+TA films exhibit long‐range molecular packing that is uniformly distributed throughout the film, which is conducive to efficient charge transport and suppresses bimolecular charge recombination. The optimizing active layer morphology achieved in TBB+TA films demonstrates that the crystallinity of the thin film could be improved through the addition of TBB and TA, as confirmed by GIWAXS, AFM, and TEM analysis.

**Figure 5:**
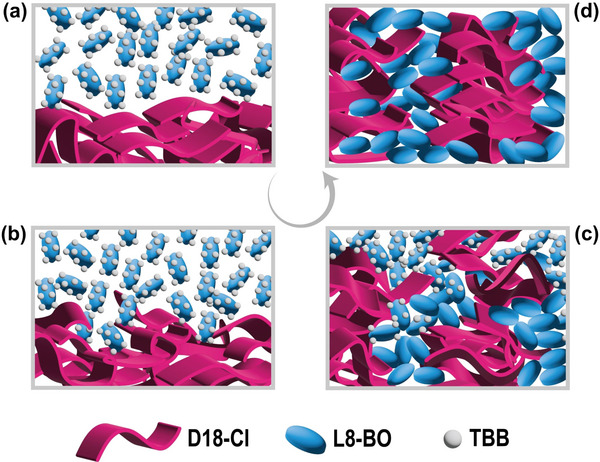
Schematic illustration of the distinction status from solution to the thin film.

According to the above results and discussions, we inferred a schematic illustration of the film evolution mechanism with the sequential deposition processing method in combination with solid additive TBB. As presented in Figure 5a, the donor layer is formed with face‐on aggregations before spin‐coating of the acceptor solution. Because of the penetration of the solvent, the donor and acceptor molecules undergo a mutual diffusion process (**Figure** **5**b) followed by a gradual aggregate formation (Figure 5c). With annealing at 75 °C for five minutes, the TBB additive evaporated completely, and the post‐treatment improved the homogeneous distribution of the crystalline domains and promoted the phase purity with clear vertical phase separation. As illustrated in Figure 5d, adding TBB and further thermal treatment can optimize the morphology by finely enhancing the acceptor crystallinity. At the same time, the relatively independent diffusion behavior of the upper small molecules leads to stronger crystallization in this strategy. In addition to the morphological changes, the improved active layer stability is noticed. We monitored the performance fluctuation with storage time to investigate the devices' stability, and the *V*_OC_, *J*_SC_, FF, and PCE were measured. As illustrated in Figure S11 (Supporting Information), TBB+TA devices still retain 92% of the initial PCE after 240 h.

## 3 Conclusion

In summary, we systematically investigated the impact of a novel volatile solid additive, TBB, on the performance of the OSCs composed of D18‐Cl and L8‐BO, fabricated with a two‐step deposition approach. We found that the addition of TBB, combined with further thermal treatment, could result in long‐range molecular packing and bi‐continuous donor‐acceptor network homogenously in the thin film, leading to increased thin film crystallinity and favorable donor‐acceptor distribution in the vertical direction. Therefore, the optimal active layer morphology is beneficial for the charge transport properties and reduces the bimolecular charge recombination. As a result, the TBB+TA OSCs achieved a champion PCE of 18.5% (18.1% averaged), with a *V*_OC_ of 910 mV, *J*_SC_ of 26.3 mA cm^−2^, and FF of 77.2%, outperforming its control devices (17.2%, 16.7% averaged). The improved device performance was associated with increased exciton generation rate, charge carrier mobility, charge carrier lifetime, and reduced bimolecular charge recombination. Our results provide insights into the design and selection of volatile solid additives for efficient non‐fullerene OSCs.

## Conflict of Interest

The authors declare no conflict of interest.

## Supporting information

Supporting Information

## Data Availability

The data that support the findings of this study are available from the corresponding author upon reasonable request.
