# Supplementary material for: Vertical Phase Regulation with 1,3,5‐Tribromobenzene Leads to 18.5% Efficiency Binary Organic Solar Cells
Source: Adv Sci (Weinh). 2023 Jul 9;10(26):2303150. doi: 10.1002/advs.202303150 (PMC10502666; doi:10.1002/advs.202303150)
Supplement: Supplementary file 1 — Supporting Information [file ADVS-10-2303150-s001.pdf]

ADVANCED SCIENCE
Open Access

Supporting Information

for *Adv. Sci.*, DOI 10.1002/advs.202303150

Vertical Phase Regulation with 1,3,5-Tribromobenzene Leads to 18.5% Efficiency Binary Organic Solar Cells

Chaofeng Zhu, Sein Chung, Jingjing Zhao, Yuqing Sun, Bin Zhao, Zhenmin Zhao, Seunghyun Kim, Kilwon Cho and Zhipeng Kan**

Supporting Information

Vertical Phase Regulation with 1,3,5-Tribromobenzene Leads to 18.5% Efficiency Binary Organic Solar Cells

Chaofeng Zhu, Sein Chung, Jingjing Zhao, Yuqing Sun, Bin Zhao, Zhenmin Zhao, Seunghyun Kim, Kilwon Cho, and Zhipeng Kan**

C. Zhu, J. Zhao, Y. Sun, B. Zhao, Z. Zhao, Prof. Z. Kan
Center on Nanoenergy Research, Guangxi Colleges and Universities Key Laboratory of Blue Energy and Systems Integration, Carbon Peak and Neutrality Science and Technology Development Institute, School of Physical Science & Technology, Guangxi University, Nanning 530004, China
E-mail: kanzhipeng@gxu.edu.cn
S. Chung, S. Kim, Prof. K. Cho
Department of Chemical Engineering, Pohang University of Science and Technology, Pohang 37673, South Korea
E-mail: kwcho@postech.ac.kr
Prof. Z. Kan
State Key Laboratory of Featured Metal Materials and Life-cycle Safety for Composite Structures, Nanning 530004, China

Contents

1. Experimental Materials 3
2. Fabrication of OSC Devices 3
3. Device Measurement 4
4. UV-Vis Absorbance and Photoluminescence (PL) Measurements 5
5. Fourier-Transform Infrared (FTIR) Spectroscopy Measurement 7
6. Dark Current Density-Voltage (*J*-*V*) Measurement 7
7. Carrier Extraction by Linearly Increasing Voltage (CELIV) Measurement 8
8. Transient Photovoltage (TPV) and Transient Photocurrent (TPC) Measurements 9
9. Film-Depth-Dependent Light Absorption Spectroscopy (FLAS) Measurement 9
10. Time-of-Fight Secondary Ion Mass Spectrometry (TOF-SIMS) Measurement 10
11. Near-Edge X-ray Absorption Fine-Structure (NEXAFS) Measurement 10
12. Transmission Electron Microscopy (TEM) Measurement 11
13. Grazing Incidence Wide-Angle X-ray Scattering (GIWAXS) Measurement 12
14. Atomic Force Microscopy (AFM) Measurement 13
15. Device Stability Data 13
16. References 14

1. Experimental Materials

Unless stated otherwise, all the chemical reagents, solvents and additives used were obtained commercially and were used without further purification. The polymer donor D18-Cl was purchased from eFlexPV Inc. L8-BO and PDIN were purchased from Solarmer Inc. PEDOT:PSS (CLEVIOSTM P VP AI 4083, Heraeus, Germany) was purchased from Xi'an Polymer Light Technology Corp.

D18-Cl [1]

Poly[(2,6-(4,8-bis(5-(2-ethylhexyl-4-chloro-2-thienyl)-benzo[1,2-b:4,5-b′]dithiophene))-alt-(2-butyloctyl)thiophen-2-yl)-8-(4-(2-butyloctyl)-5-methylthiophen-2-yl)dithieno [3′,2′:3,4;2″,3″:5,6]benzo[1,2-c][1,2,5]thiadiazole)]

L8-BO [2]

2,2′-((2Z,2′Z)-((3,9-bis(2-butyloctyl)-12,13-bis(2-ethylhexyl)-12,13-dihydro-[1,2,5] thiadiazolo[3,4-e]thieno[2″,3″:4′,5′]thieno[2′,3′:4,5]pyrrolo[3,2-g]thieno[2′,3′:4,5]thieno[3,2-b]indole-2,10-diyl)bis(methaneylylidene))bis(5,6-difluoro-3-oxo-2,3-dihydro-1H-indene-2,1-diylidene))dimalononitrile

PDIN [3]

2,9-bis[(3-dimethylamino)propyl]-anthra[2,1,9-def:6,5,10-d′e′f′]diisoquinoline-1,3,8,10 (2H,9H)-tetrone

2. Fabrication of OSC Devices

The OSC devices with a normal architecture (indium tin oxide (ITO)/PEDOT:PSS/Active Layer/PDIN/Ag) were prepared with the following procedures. Prior to device fabrication, ITO-coated glass (1.5 cm × 1.5 cm, sheet resistance = 15 Ω $square^{-1}$) substrates were subsequently cleaned in an ultrasonic bath with detergent, deionized water, acetone, deionized water, and isopropyl alcohol.

After being treated in an ultraviolet-ozone chamber for 30 minutes, a 30 nm PEDOT:PSS (CLEVIOSTM P VP AI 4083, Heraeus, Germany) thin film was fabricated on the cleaned ITO substrates by spin-coating method at 4500 round per minute (rpm) for 20 seconds, and then annealed at 150 °C for 20 minutes in ambient conditions. After annealing treatment, the ITO substrates coated PEDOT:PSS films were transferred to a high-purity nitrogen-filled glove box to fabricate photoactive layers. All solutions were prepared in a nitrogen-filled

glovebox using the polymer donor (D18-Cl), acceptor (L8-BO), solid additive (TBB), and PDIN. For the sequential deposition (SD) spin-coating photoactive layer, the donor D18-Cl and acceptor L8-BO were dissolved into chloroform (CF) with a concentration of 6.5 and 8.5 mg mL^{-1}, respectively, and then heated for 2 hours at 45 ℃.

Firstly, the D18-Cl was deposited on PEDOT:PSS film as front layer at 5000 rpm for 30 seconds and then the L8-BO solution was spin-coated on the top of D18-Cl film at 4000 rpm for 30 seconds. Notably, the D18-Cl/L8-BO (Control) film was prepared without adding the additive TBB and without any post-treatment. The D18-Cl/L8-BO (TBB) film was prepared by adding the additive TBB (15 mg mL^{-1}), which was added only to the receptor L8-BO CF-solution. No post-treatment was applied to this film. The D18-Cl/L8-BO (TBB+TA) film was prepared with the addition of the additive TBB (15 mg mL^{-1}, added only to the receptor L8-BO CF-solution), followed by annealing post-treatment at 75 ℃ for 5 minutes. After that, PDIN solution (2.0 mg mL^{-1} in methanol with 0.3 vol% acetic acid) was spin-coated on the top of photoactive layers at 5000 rpm for 20 seconds. Subsequently, the cathode of Ag was deposited by thermal evaporation with a shadow mask under 1×10^{-6} Torr and the thickness of 100 nm was monitored by a quartz crystal microbalance. The photoactive effective area of OSCs is about 4 mm^2, which is defined by the overlap of ITO anode and Ag cathode.

3. Device Measurement

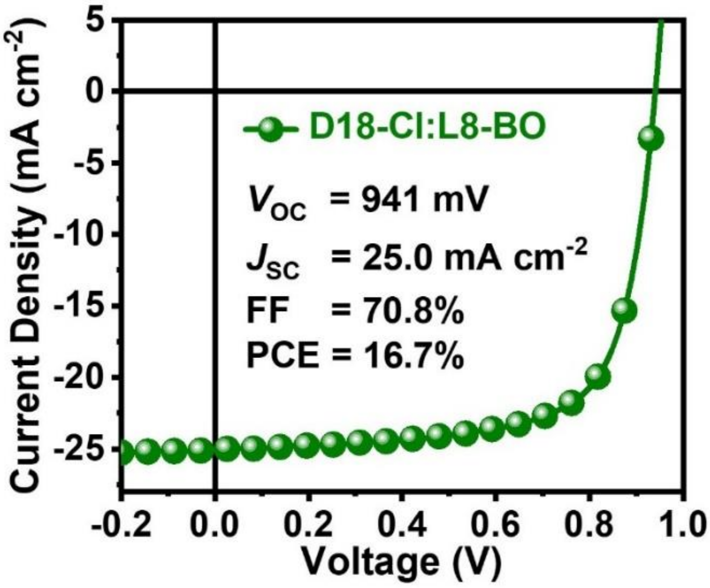

Figure S1. Current density-voltage (*J-V*) characteristic of OSC device composed of D18-Cl:L8-BO under simulated AM 1.5 G irradiation (100 mW cm^{-2}).

Current density-voltage (*J-V*) measurement was performed via a XES-50S1 (SAN-EI Electric Co., Ltd.) solar simulator (AAA grade) whose intensity was calibrated by a certified standard silicon solar cell (SRC-2020, Enlitech) under illumination of AM 1.5 G simulated

irradiance (100 mW cm^{-2}). The AM 1.5 G light source with a spectral mismatch factor of 1.01 was calibrated by the National Institute of Metrology. The intensity of the AM 1.5 G spectra was calibrated by a certified standard silicon solar cell (SRC-2020, Enlitech) calibrated by the National Institute of Metrology. The *J-V* curves of small-area devices were measured in forwarding scan mode (from -0.2 V to 1.2 V) with a scan step length of 0.02 V. The external quantum efficiency (EQE) was measured by a certified incident photon to electron conversion (IPCE) equipment (QE-R) from Enli Technology Co., Lt. The light intensity at each wavelength was calibrated using a standard monocrystalline Si photovoltaic cell.

Photovoltaic parameters of D18-Cl/L8-BO-based OSCs versus the concentration of TBB under AM 1.5 G simulated irradiance (100 mW cm^{-2}). The TBB processed device is tolerant to additive concentration, the device can maintain 18.5% champion efficiency with a TBB concentration of 15 mg mL^{-1}.

Table S1. Optimization of TBB content in L8-BO for SD OSCs. Average values with standard deviation (in parenthesis) were obtained from 10 independent devices.

Additive	V_{OC} [mV]	J_{SC} [mA cm^{-2}]	FF [%]	PCE [%]
without TBB	945	25.81	70.62	17.22 (16.73 ± 0.34)
with TBB, 5 mg mL^{-1}	919	25.56	74.94	17.66 (17.24 ± 0.28)
with TBB, 10 mg mL^{-1}	915	26.11	75.89	18.25 (17.92 ± 0.23)
with TBB, 14 mg mL^{-1}	909	26.32	76.98	18.43 (18.06 ± 0.29)
with TBB, 15 mg mL^{-1}	910	26.30	77.21	18.49 (18.11 ± 0.31)
with TBB, 16 mg mL^{-1}	900	26.40	76.17	18.10 (17.81 ± 0.27)
with TBB, 20 mg mL^{-1}	889	26.41	75.72	17.80 (17.64 ± 0.19)

4. UV-Vis Absorbance and Photoluminescence (PL) Measurements

UV-Vis absorbance spectra of different thin films were recorded on a UV-5200PC UV-Vis spectrophotometer of Shanghai Metash Instruments Co., Ltd. The photoluminescence of the films was measured by a FLS1000 equipped with an integrating hemisphere at an excitation wavelength of 550 nm and 780 nm from Edinburgh Instruments Co., Ltd.

Fabrication of the active layer thin films for the UV-Vis absorbance and photoluminescence (PL) measurements below: the glass (1.5 cm × 1.5 cm) substrates were cleaned in an ultrasonic bath with detergent, deionized water, acetone, deionized water, and isopropyl alcohol. The glass substrates were blown dry with a nitrogen gun. After being

treated in an ultraviolet-ozone chamber for 30 minutes, the D18-Cl was deposited on the glass substrates as front layer at 5000 rpm for 30 seconds and then the L8-BO solution was spin-coated on the top of D18-Cl film at 4000 rpm for 30 seconds.

→The D18-Cl/L8-BO (Control) film was prepared without adding the additive TBB and without any post-treatment.

→The D18-Cl/L8-BO (TBB) film was prepared by adding the additive TBB, which was added only to the receptor L8-BO CF-solution. No post-treatment was applied to this film.

→The D18-Cl/L8-BO (TBB+TA) film was prepared with the addition of the additive TBB (15 mg mL^{-1}, added only to the receptor L8-BO CF-solution), followed by annealing post-treatment at 75 °C for 5 minutes.

→The neat D18-Cl film was prepared without adding the additive TBB and without any post-treatment. The D18-Cl was deposited on the glass substrates at 5000 rpm for 30 seconds.

→The neat L8-BO (Control) film was prepared without adding the additive TBB and without any post-treatment. The L8-BO was deposited on the glass substrates at 4000 rpm for 30 seconds.

→The L8-BO (TBB) film was prepared by adding the additive TBB, which was added only to the receptor L8-BO CF-solution. The L8-BO (TBB) was deposited on the glass substrates at 4000 rpm for 30 seconds. No post-treatment was applied to this film.

→The L8-BO (TBB+TA) film was prepared with the addition of the additive TBB (added only to the receptor L8-BO CF-solution; The L8-BO (TBB) was deposited on the glass substrates at 4000 rpm for 30 seconds.), followed by annealing post-treatment at 75 °C for 5 minutes.

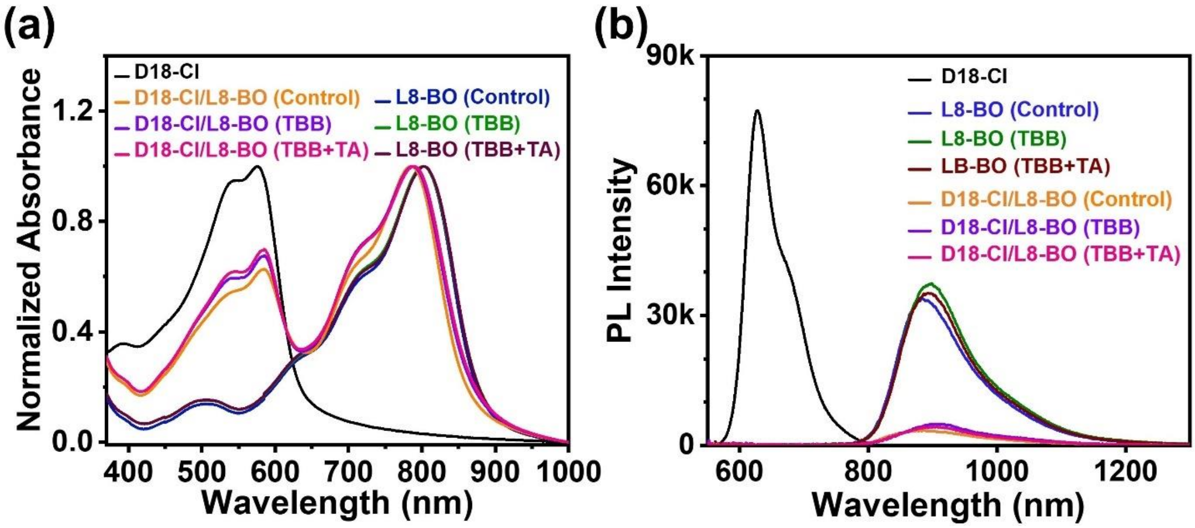

Figure S2. (a) Absorbance and (b) photoluminescence spectra of the neat and LBL-processed films.

5. Fourier-Transform Infrared (FTIR) Spectroscopy Measurement

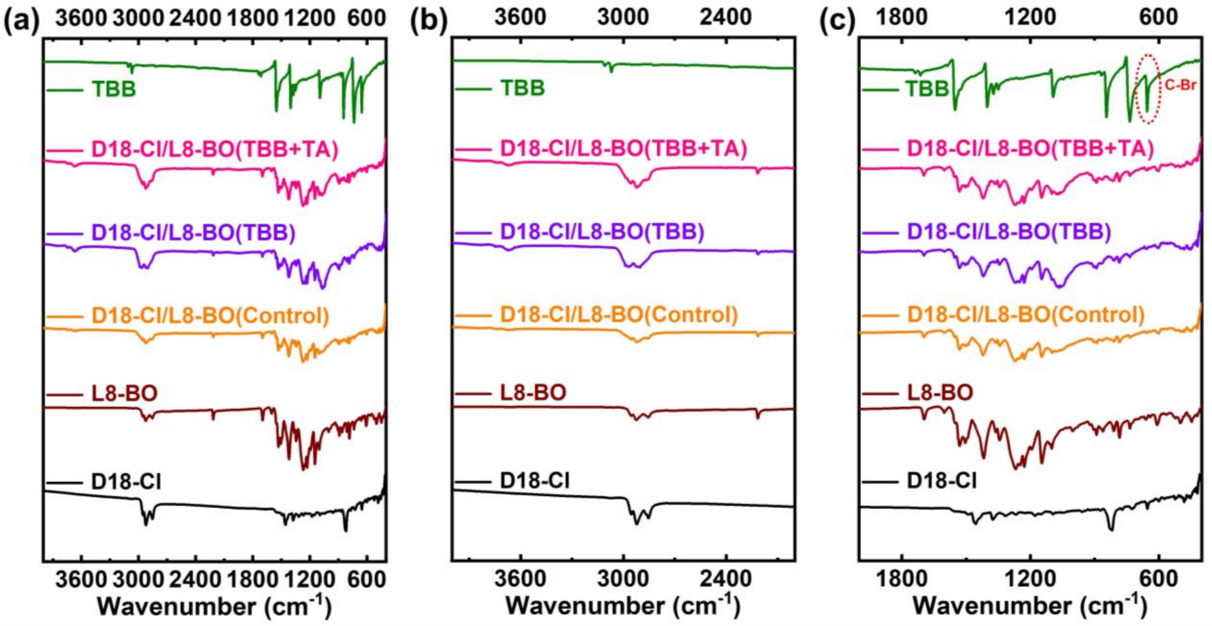

Figure S3. Infrared spectra (IR) plots of D18-Cl, L8-BO, TBB and D18-Cl/L8-BO (Control) film, and TBB-processed D18-Cl/L8-BO with or without thermal annealing (TA).

6. Dark Current Density-Voltage (*J*-*V*) Measurement

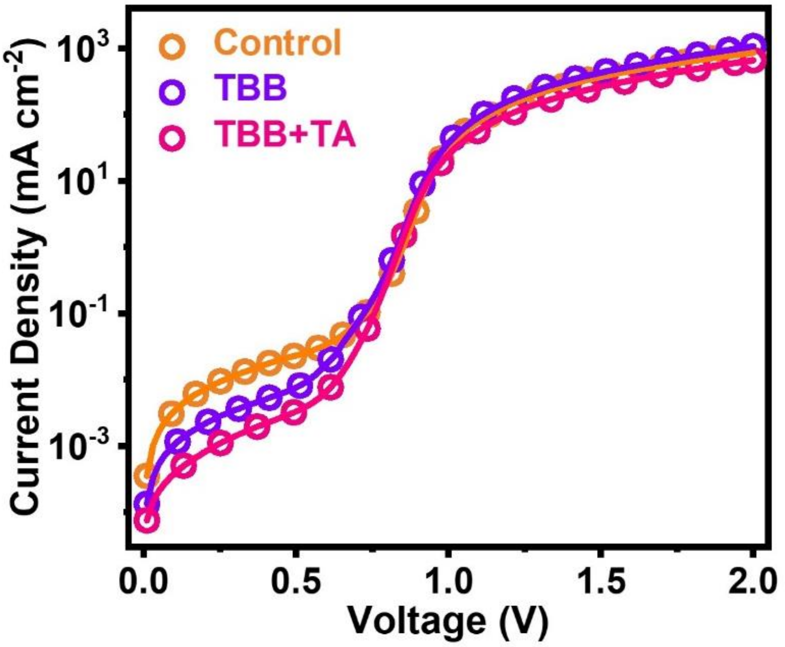

Figure S4. Dark current of the fabricated D18-Cl/L8-BO OSCs.

Mathematically this model is represented by the relationship:

$$J = J_0 \left\{ exp \left[\frac{e(V - JR_S)}{nk_BT} \right] - 1 \right\} + \frac{V - JR_S}{R_{sh}} - J_{ph} \qquad \text{(Equation)}$$

where J_0 is the reverse bias saturation current density, e is the elementary charge, V is the cell voltage, J is the current density, R_s is the series resistance, n is the diode ideality factor, k_B is Boltzmann's constant, T is temperature, and R_{sh} is the equivalent circuit shunt, or parallel resistance. J_{ph} is the photocurrent generated by the cell before recombination losses. According to the original p-n junction theory of Shockley, J_0 represents the saturation current density at reverse bias for an ideal *p-n* junction diode. For this ideal diode, n = 1. [4, 5]

Table S2. The analytical parameters of the positive bias part were fitted with the diode equation.

Device (D18-Cl/L8-BO)	n	R_s (Ω·m^2)	R_{sh} (Ω·m^2)
Control	1.573±0.016	$1.087\times10^{-4}\pm2.558\times10^{-6}$	2.475±0.057
TBB	1.697±0.020	$0.909\times10^{-4}\pm2.146\times10^{-6}$	8.352±0.201
TBB+TA	1.553±0.013	$1.575\times10^{-4}\pm4.002\times10^{-6}$	20.508±0.573

7. Carrier Extraction by Linearly Increasing Voltage (CELIV) Measurement

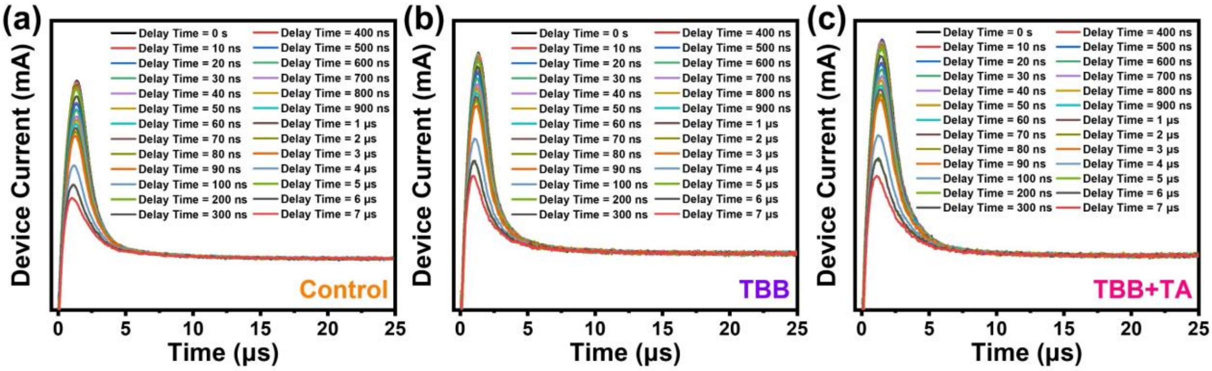

Figure S5. Photo-CELIV measurements on the optimized D18-Cl/L8-BO (Control) (a), D18-Cl/L8-BO (TBB) (b), and D18-Cl/L8-BO (TBB+TA) (c) devices for different delay times between the light pulse and the extraction voltage ramp.

According to Mozer et al.'s recombination model, carrier density $n(t)$ over time can be expressed as: [6, 7]

$$n(t) = \frac{n_0}{1+\left(\frac{t}{\tau_b}\right)^{\gamma}} \qquad \text{Equation (1)}$$

where $n(t)$ is the charge density at time t, n_0 is the initial charge density, τ_b is the recombination lifetime, and γ is the dispersion parameter. According to ref. γ = 1 for trap-free bimolecular recombination and is γ < 1 for the dispersive bimolecular recombination.

In the dispersive bimolecular recombination, the decay of carrier density is given by:

$$\beta(\mathrm{t}) = -\frac{dn(t)/d(t)}{n^2(t)} \qquad \text{Equation (2)}$$

where $n(t)$ is the carrier density and $\beta(\mathrm{t})$ is dispersive bimolecular recombination rate at a delay time t. Substituting Equation (1), the bimolecular recombination rate $\beta(\mathrm{t})$ can also be expressed as: [8, 9]

$$\beta(\mathrm{t}) = (1/\tau_b)\gamma {n_0}^{-1}(t/\tau_b)^{\gamma-1} \qquad \text{Equation (3)}$$

The resulting $\beta(\mathrm{t})$ can be calculated from the fitting parameters n_0, τ_b and γ using Equation (3).

Table S3. Dispersive bimolecular recombination fitting parameters for the optimized OSCs.

Device (D18-Cl/L8-BO)	n_0 (×10^{16} cm^{-3})	τ_b (10^{-5} s)	β (×10^{-13} cm^3 s^{-1})
Control	6.933	2.763	5.220
TBB	7.804	2.732	4.690
TBB+TA	9.708	2.582	3.989

8. Transient Photovoltage (TPV) and Transient Photocurrent (TPC) Measurements

TPV and TPC measurements were obtained on an all-in-one characterization platform Paios developed by Fluxim AG, Switzerland.

9. Film-Depth-Dependent Light Absorption Spectroscopy (FLAS) Measurement

The film-depth-dependent light absorption spectroscopy (FLAS) was acquired by a home-made in-situ setup using a spectrometer equipped with a soft plasma-ion source (PU100, Shaanxi Puguang Weishi Co. Ltd.). The power-supply for generating the soft ionic source is 100 W with input oxygen pressure ~10 Pa.

FLAS was performed by a homemade instrument as previously reported. [10] A self-developed soft plasma source generated by glow-discharging of low-pressure oxygen was used to incrementally etch the film surface without damage to the materials underneath the surface, which is in-situ monitored by a spectrometer. FLAS is extracted from the evolution of the light absorption spectra during soft plasma etching. From the evolution of the spectra and the Beer-Lambert's Law, film-depth-dependent absorption spectra were extracted. A self-developed capacitive-coupled plasma generator was utilized to generate soft ionic plasma,

which etched the film without damage to the underneath materials. This warranted the reliable measurement of the light absorption spectra of the films during etching. The soft plasma etching procedure was performed with N_2 gas pressure < 30 Pa by Diener Femto plasma cleaner. The soft plasma etching is harmless to the beneath layers, especially the optical property. The film-depth-dependent exciton generation is obtained upon the modified optical transmission matrix method, taking film-depth-dependent light absorption spectra and optical interference into simulation. The detail of the optical modeling is available in the literature published by Lu et a1. [11-22]

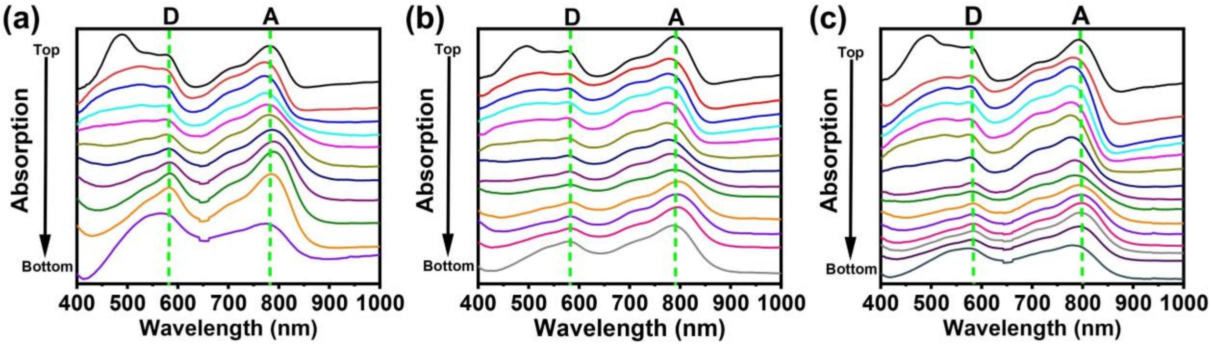

Figure S6. The film-depth-dependent light absorption spectroscopy (FLAS) spectra of the D18-Cl/L8-BO (Control) (a), D18-Cl/L8-BO (TBB) (b), and D18-Cl/L8-BO (TBB+TA) (c) films.

10. Time-of-Fight Secondary Ion Mass Spectrometry (TOF-SIMS) Measurement

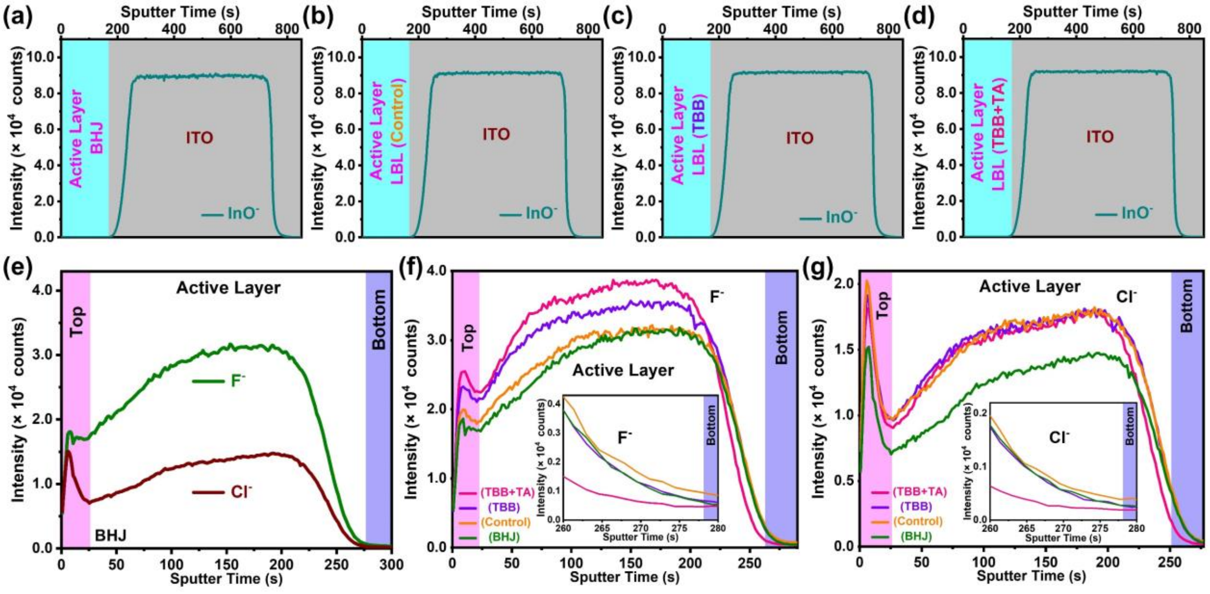

Figure S7. TOF-SIMS ion yield of F^-, Cl^-, and InO^- as a function of sputtering time for the BHJ, Control, TBB, and TBB+TA blend films composed of D18-Cl and L8-BO.

11. Near-Edge X-ray Absorption Fine-Structure (NEXAFS) Measurement

NEXAFS is usually used to study the chain orientation of polymer molecules, as well as the composition near the surface of thin films, and it studies the transition between the core state (e.g., carbon 1s level) to unoccupied molecular orbitals (π* and σ* orbitals) of molecules. During NEXAFS measurements, a controlled X-ray beam is induced to bombard the surface of the film. The X-ray radiation can be absorbed when it is polarized in the direction of the transition dipole moment (TDM) of the molecular electron orbitals. Electron signals can be collected for element-specific information. Electron yield signals from NEXAFS include total electron yield (TEY) (with a depth sensitivity of 10 nm) and Auger electron yield (AEY) (depth sensitivity of 1~2 nm), which can provide quantification of the mass composition of the film surface and buried interface after delamination to expose the buried interface. Different spectra are obtained for materials with different orientations or compositions.

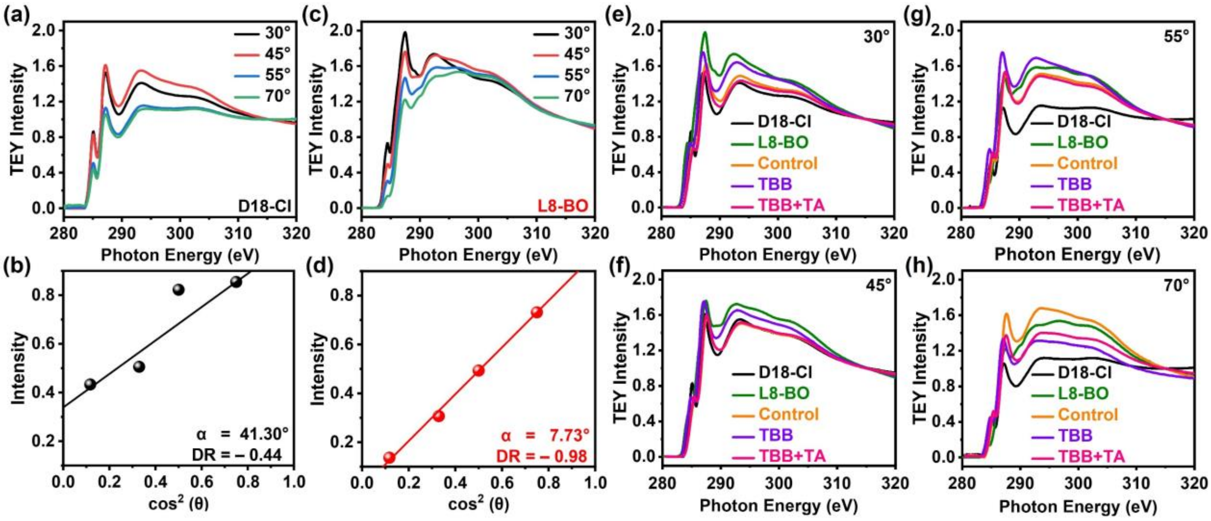

Figure S8. (a, c) Angular dependence of carbon K-edge NEXAFS spectra in total electron yield (TEY) detection mode for neat D18-Cl (a) and neat L8-BO (c) films made from spin-coating method, with X-ray beam at 30°, 45°, 55°, 70° (grazing incident) related to the sample surface. (b, d) Orientation analysis for neat films. (e-h) Angular dependence of carbon K-edge NEXAFS spectra in total electron yield (TEY) detection mode for five films made from spin-coating method, with X-ray beam at 30°, 45°, 55°, 70° (grazing incident) related to the sample surface, respectively.

12. Transmission Electron Microscopy (TEM) Measurement

Using PEDOT:PSS as a sacrificial layer on a glass substrate, the spun-cast film was peeled from the substrate by dissolving the PEDOT:PSS layer in water and then transferred

onto a 50-mesh copper grid (Electron Microscopy China), and viewed by a spherical aberration-corrected electron microscope (Titan ETEM G2 E-Twin) operated at an acceleration voltage of 300 keV.

13. Grazing Incidence Wide-Angle X-ray Scattering (GIWAXS) Measurement

Grazing incidence wide-angle X-ray scattering (GIWAXS) was measured at 3C and 9A beam lines of the Pohang Light Source II (PLS-II), Pohang Accelerator Laboratory, Korea. GIWAXS was carried out to investigate the molecular packing and molecular orientation in the thin film. The samples were prepared on Si substrates using the identical conditions with the device fabrication. The π-π stacking distance (d-spacing) obtained by the equation of d-spacing = 2π/q, in which q is the corresponding x-coordinate of diffraction peak. The crystalline coherence length (CCL) was calculated quantitatively by using the Scherrer equation: CCL = $2\pi K/\Delta q$, in which K is a form factor (K = 0.9), and Δq is the full-width at half-maximum (FWHM) of the diffraction peak.

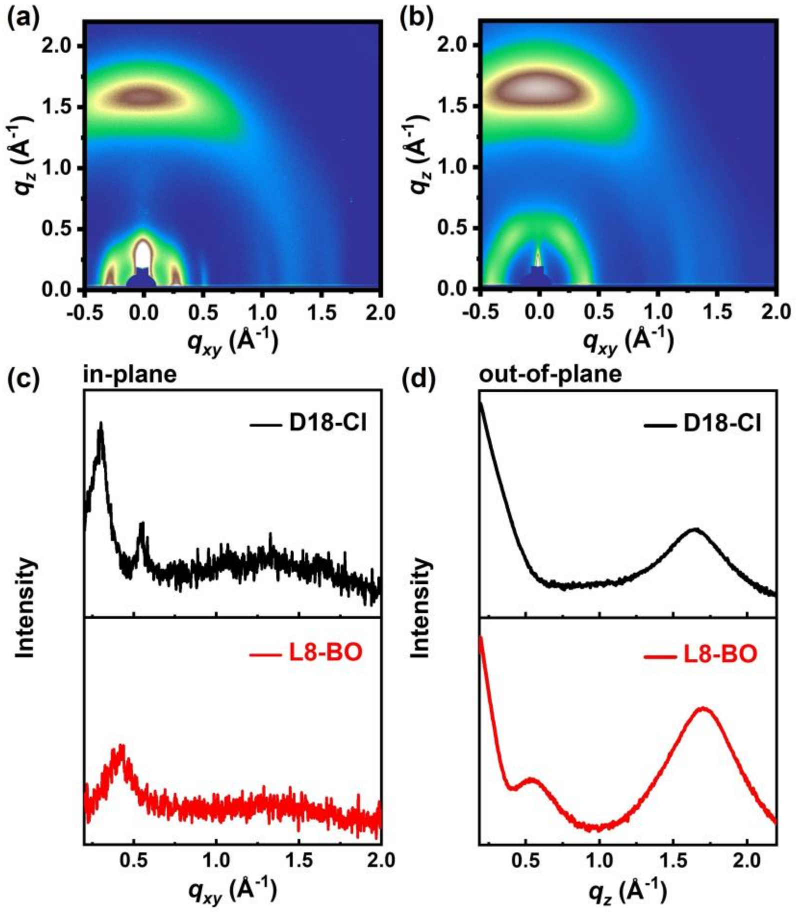

Figure S9. (a, b) 2D GIWAXS patterns of neat films: (a) D18-Cl and (b) L8-BO. (c, d) GIWAXS intensity profiles of two neat films along the corresponding in-plane (c) and out-of-plane (d) line-cuts.

Table S4. In-plane (IP) and out-of-plane (OOP) parameters: peak location, d-spacing, FWHM, and crystal coherence length (CCL) extracted from the 2D GIWAXS of five films.

System	Peak	Peak Location (Å^{-1})	d-Spacing (Å)	FWHM (Å^{-1})	CCL (Å)
D18-Cl	IP (100)	0.295	21.28	0.074	76.48
	OOP (010)	1.640	3.83	0.259	21.84
L8-BO	IP (100)	0.411	15.30	0.195	29.06
	OOP (200)	0.533	11.79	0.191	29.66
	OOP (010)	1.700	3.70	0.289	19.57
D18-Cl/L8-BO (Control)	IP (100)	0.321	19.58	0.114	49.56
	OOP (010)	1.677	3.75	0.277	20.39
D18-Cl/L8-BO (TBB)	IP (100)	0.311	20.18	0.074	76.06
	OOP (010)	1.684	3.73	0.288	19.61
D18-Cl/L8-BO (TBB+TA)	IP (100)	0.312	20.16	0.063	89.41
	OOP (010)	1.697	3.70	0.270	20.96

14. Atomic Force Microscopy (AFM) Measurement

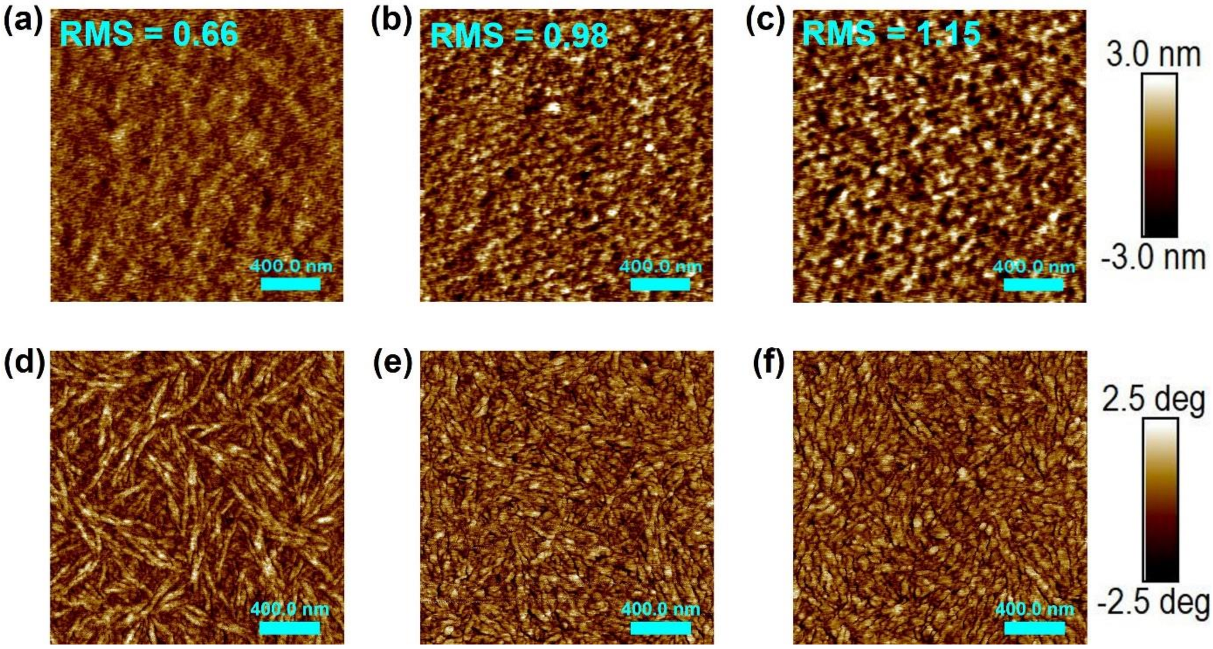

Figure S10. Atomic force microscopy (AFM) height and phase images of (a, d) D18-Cl/L8-BO (Control), (b, e) D18-Cl/L8-BO (TBB), and (c, f) D18-Cl/L8-BO (TBB+TA) films.

15. Device Stability Data

To investigate the stability capability of four type OSC devices, the performance fluctuation with storage time was operated, and the V_{OC}, J_{SC}, FF, and PCE were traced for about 240 h. Apparently, stabilized V_{OC} is observed in four type OSCs, whereas different J_{SC} and FF losses are found and further led to the degradation in PCE. Compared with the PCE of BHJ device decreasing to 86.4%, TBB+TA device still retains a PCE of 92%. Meanwhile, the FF of BHJ device and TBB+TA device after 240 h is 94% and 97%, respectively. Therefore, the stability of TBB+TA device outperforms the BHJ device.

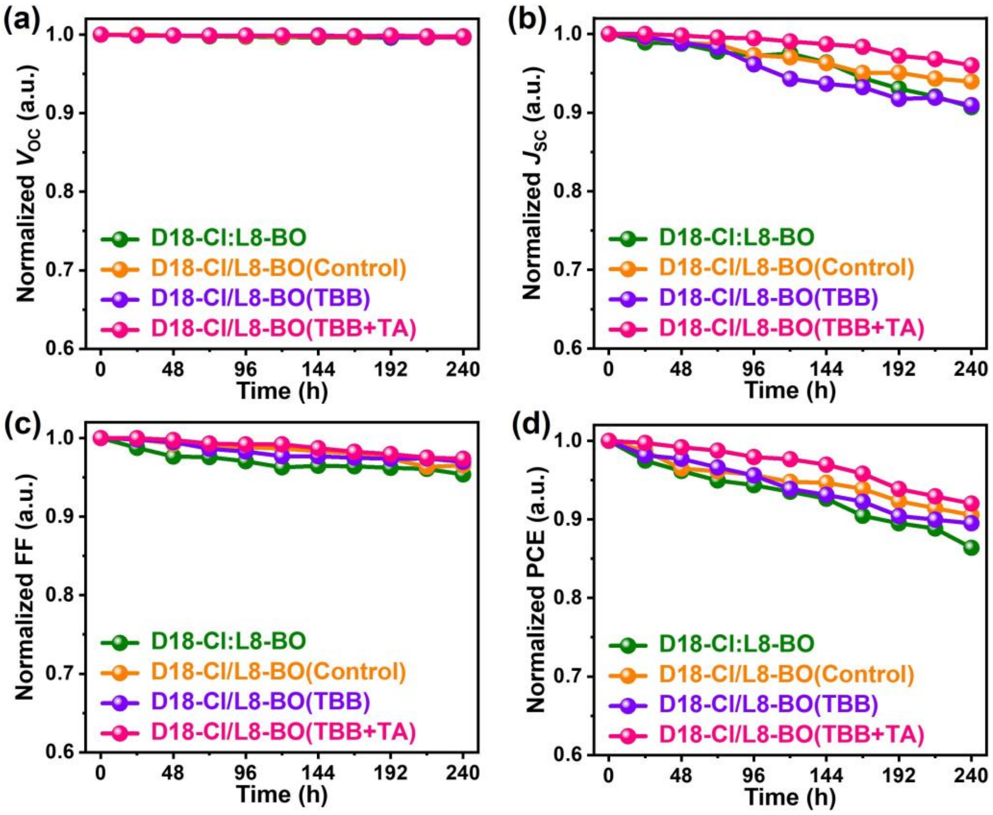

Figure S11. Device stability. Normalized parameters of (a) V_{OC}, (b) J_{SC}, (c) FF, and (d) PCE for D18-Cl:L8-BO, D18-Cl/L8-BO (Control), D18-Cl/L8-BO (TBB), and D18-Cl/L8-BO (TBB+TA) devices with conventional structure in a dry nitrogen atmosphere without encapsulation.

16. References

[1] A., Zeng, X. Ma, M. Pan, Y. Chen, R. Ma, H. Zhao, J. Zhang, H. K. Kim, A. Shang, S. Luo, I. C. Angunawela, Y. Chang, Z. Qi, H. Sun, J. Y. L. Lai, H. Ade, W. Ma, F. Zhang, H. Yan, *Adv. Funct. Mater.* **2021**, *31*, 2102413.

[2] L. Zhu, M. Zhang, J. Xu, C. Li, J. Yan, G. Zhou, W. Zhong, T. Hao, J. Song, X. Xue, Z. Zhou, R. Zeng, H. Zhu, C.-C. Chen, R. C. I. Mackenzie, Y. Zou, J. Nelson, Y. Zhang, Y. Sun, F. Liu, *Nat. Mater.* **2022**, *21*, 656.

[3] Y. Yan, X. Zhou, F. Zhang, J. Zhou, T. Lin, Y. Zhu, D. Xu, X. Ma, Y. Zou, X. Li, *J. Mater. Chem. A* **2022**, *10*, 23124.

[4] J. D. Servaites, S. Yeganeh, T. J. Marks, M. A. Ratner, *Adv. Funct. Mater.* **2010**, *20*, 97.

[5] J. D. Servaites, M. A. Ratner, T. J. Marks, *Energy Environ. Sci.* **2011**, *4*, 4410.

[6] A. Pivrikas, G. Juška, A. J. Mozer, M. Scharber, K. Arlauskas, N. S. Sariciftci, H. Stubb, R. Österbacka, *Phys. Rev. Lett.* **2005**, *94*, 176806.

[7] A. J. Mozer, G. Dennler, N. S. Sariciftci, M. Westerling, A. Pivrikas, R. Österbacka, G. Juška, *Phys. Rev. B* **2005**, *72*, 035217.

[8] J. Guo, H. Ohkita, S. Yokoya, H. Benten, S. Ito, *J. Am. Chem. Soc.* **2010**, *132*, 9631.

[9] Y. Liu, Y. Gao, B. Xu, P. H. M. Van Loosdrecht, W. Tian, *Org. Electron.* **2016**, *38*, 8.

[10] Z. Wang, Y. Hu, T. Xiao, Y. Zhu, X. Chen, L. Bu, Y. Zhang, Z. Wei, B. Xu, G. Lu, *Adv. Optical Mater.* **2019**, *7*, 1900152.

[11] L. Bu, M. Hu, W. Lu, Z. Wang, G. Lu, *Adv. Mater.* **2018**, *30*, 1704695.

[12] J. Wang, J. Zhang, Y. Xiao, T. Xiao, R. Zhu, C. Yan, Y. Fu, G. Lu, X. Lu, S. R. Marder, X. Zhan, *J. Am. Chem. Soc.* **2018**, *140*, 9140.

[13] W. Yang, W. Wang, Y. Wang, R. Sun, J. Guo, H. Li, M. Shi, J. Guo, Y. Wu, T. Wang, G. Lu, C. J. Brabec, Y. Li, J. Min, *Joule* **2021**, *5*, 1209.

[14] R. Sun, Y. Wu, J. Guo, Y. Wang, F. Qin, B. Shen, D. Li, T. Wang, Y. Li, Y. Zhou, G. Lu, Y. Li, Jie Min, *Energy Environ. Sci.* **2021**, *14*, 3174.

[15] Y. Zheng, R. Sun, M. Zhang, Z. Chen, Z. Peng, Q. Wu, X. Yuan, Y. Yu, T. Wang, Y. Wu, X. Hao, G. Lu, H. Ade, J. Min, *Adv. Energy Mater.* **2021**, *11*, 2102135.

[16] F.-Z. Cui, Z.-H. Chen, J.-W. Qiao, T. Wang, G.-H. Lu, H. Yin, X.-T. Hao, *Adv. Funct. Mater.* **2022**, *32*, 2200478.

[17] S. Li, Q. Fu, L. Meng, X. Wan, L. Ding, G. Lu, G. Lu, Z. Yao, C. Li, Y. Chen, *Angew. Chem. Int. Ed.* **2022**, *61*, e202207397.

[18] H. Fu, Z. Peng, Q. Fan, F. R. Lin, F. Qi, Y. Ran, Z. Wu, B. Fan, K. Jiang, H. Y. Woo, G. Lu, H. Ade, A. K.-Y. Jen, *Adv. Mater.* **2022**, *34*, 2202608.

[19] C. He, Y. Pan, G. Lu, B. Wu, X. Xia, C.-Q. Ma, Z. Chen, H. Zhu, X. Lu, W. Ma, L. Zuo, H. Chen, *Adv. Mater.* **2022**, *34*, 2203379.

[20] Y. Wei, Z. Chen, G. Lu, N. Yu, C. Li, J. Gao, X. Gu, X. Hao, G. Lu, Z. Tang, J. Zhang, Z. Wei, X. Zhang, Hui Huang, *Adv. Mater.* **2022**, *34*, 2204718.

[21] Y. Cai, Q. Li, G. Lu, H. S. Ryu, Y. Li, H. Jin, Z. Chen, Z. Tang, G Lu, X Hao, H. Y. Woo, C. Zhang, Y. Sun, *Nat. Commun.* **2022**, *13*, 2369.

[22] X. Yang, R. Sun, Y. Wang, M. Chen, X. Xia, X. Lu, G. Lu, J. Min, *Adv. Mater.* **2023**, *35*, 2209350.
